# Mut zur Lücke. Verantwortungszuschreibungen auf der Ebene sozialer Beziehungen als neuer Bereich der Framing-Forschung zu Gesundheitsthemen

**DOI:** 10.1007/s11616-021-00652-5

**Published:** 2021-04-22

**Authors:** Doreen Reifegerste, Annemarie Wiedicke, Linn Julia Temmann, Sebastian Scherr

**Affiliations:** 1 Fakultät für Gesundheitswissenschaften, Universität Bielefeld, Universitätsstr. 25, 33615 Bielefeld, Deutschland; 2 Texas A&M University, College Station, USA

**Keywords:** Responsibility Framing, Gesundheitskommunikation, Berichterstattung, Attribution, Soziales Netzwerk, Responsibility framing, Health communication, Media coverage, Attribution, Social networks

## Abstract

Frames der Verantwortungszuschreibung (Responsibility Frames) in der Medienberichterstattung betonen sowohl spezifische Ursachen als auch spezifische Lösungen für ein Thema. Die Forschung zum Responsibility Framing von Medieninhalten und deren Wirkungen untersucht diese Betonung bislang zumeist auf zwei verschiedenen Ebenen: der Individualebene und der gesellschaftlichen Ebene. Diese Betrachtungsweise vernachlässigt allerdings die wichtige mittlere Ebene des sozialen Umfelds der Menschen. Hier befinden sich zentrale Akteur*innen (wie Familie, Freund*innen oder Kolleg*innen), die als Ursache oder Lösung zu einem Problem beitragen können, was insbesondere bei Gesundheitsproblemen deutlich wird. Das Ziel des Beitrags ist es daher, die Ebene sozialer Beziehungen in das Konzept des Responsibility Framings zu integrieren. Dafür verknüpfen wir zentrale Elemente der sozialen Netzwerktheorie (Akteur*innen und ihre Funktionen für das Individuum) und der Attributionstheorie mit den Framing-Konzepten. Darauf aufbauend werden Konsequenzen für die zukünftige Forschung der Kommunikationswissenschaft abgeleitet.

## Abstract (en)

This article conceptualizes the role of the social network within the concept of responsibility framing. Central elements of social network theory (different actors and their functions for the individual) and attribution theory are linked with research on media frames and framing. Specifically, we integrate the social network as a distinct influence level between the level of the individual and the societal level, which have been predominantly focused in existing research on the concept of responsibility framing.

Social relationships are relevant across different communication contexts, e.g., activism or education, but their most important role might be in health communication. Health and health responsibility are significantly determined by an individual’s social relationships – both informal ones (e.g., family and friends) and formal ones (e.g., colleagues at work). Social relationships come with different functions including providing social support, shaping social norms, or influencing opinions or behaviors, and they thereofore strongly contribute to a person’s physical and mental constitution. Consequently, research in the health and social sciences acknowledges the social network as a distinct determinant for individual health. However, despite its significant impact, both news media reports about health issues and communication studies on health reporting largely disregard influences on the social network level.

This is particularly evident within research on the concept of *responsibility frames*, i.e., frames emphasizing responsibility for causes of or solutions to a health issue. Many content analytical and experimental studies on responsibility framing focus on responsibility attributions at the individual vs. societal level. Therefore, only little is known about the prevalence and effects of responsibility frames on the level of an individuals social network. Few recent studies on the responsibility framing of chronic illness, however, indicate that responsibility attributions to the social network do appear in the media coverage, and that they seem to have an effect on the recipients’ responsibility attributions. From studies investigating the effects of responsibility frames at the individual vs. societal level, we know that individual frames lead to more individualized attributions and may inhibit the recipients’ policy endorsement and lower intentions of civic engagement. It is therefore likely that frames at the social network level significantly shape attributions, opinions and behavioral intentions of the audience. In addition, attribution theory suggests that certain characteristics of an issue, such as perceived controllability, influence intentions to help individuals affected by this issue. However, existing studies have not yet examined the extent to which the framing of responsibility attributions at the network level influences social support intentions.

Overall, important factors influencing opinions and (health) behaviors, precisely factors at the social network level, have so far been largely neglected in framing research. Consequently, it is our objective to extend the concept of responsibility frames to include the level of social networks (in addition to the individual and societal level). With a three-level-model of the responsibility framing process, we demonstrate

(1) how to include responsibility attributions at the network level in the framing process (frame building, frame setting) and (2), the consequences for research in communication studies. *Frame building* describes which factors influence the emergence of journalistic frames. Research suggests that if the social network plays an important role for a specific (health) behavior, strategic communication reflects this with social appeals or by addressing relevant pro-social behaviors. Moreover, a content analysis on the responsibility framing of diabetes mellitus and depression shows that social network influences are indeed prevalent in journalistic media coverage, albeit less than individual and social influences. As compared to media frames and framing effects, research on journalists’ frames and their attributions of responsibility is very rare. Therefore, an explorative approach to journalists’ responsibility frames seems appropriate.

In the *frame setting *process, the media frames are studied as an independent variable. Thus, studies seek to answer the question whether and how media frames are reflected in the cognitive frames of the recipients—a question which is usually examined by experiments. To account for influences of the social network, both responsibility frames and the recipients’ responsibility attributions to the social network level should be considered in experimental studies.

(3) We also discuss which topics are suitable for a transfer of this new theoretical conceptualization of responsibility framing. Since responsibility frames are defined as generic (i.e., independent of specific issues), we provide a theoretical foundation for framing research that can be applied to a variety of other topics. By outlining the results of individual studies of the DFG project “Representation and Effects of Responsibility Frames for Health Topics: A Comparison of the Relevance of the Individual, the Social Network, and the Society” (DFG project number RE-3650/4-1) we want to help make our model applicable to other research topics.

## Einleitung

Soziale Beziehungen sind ein wichtiger Einflussfaktor für die Gesundheit (vgl. Valente 2015, S. 205). Dies gilt sowohl für das informelle soziale Netzwerk, wie Familie und Freund*innen, als auch für das formelle soziale Netzwerk, wie Kolleg*innen und Arbeitgeber*innen (vgl. Heaney und Israel 2008, S. 190). Die Beziehungen zu Familienmitgliedern, Freund*innen, Kolleg*innen mit ihren vielfältigen Funktionen (wie soziale Unterstützung, Meinungsbeeinflussung, soziale Kontrolle usw.) können zu einer besseren physischen und psychischen Verfassung führen (vgl. Thoits 2011, S. 145) und eine schnellere Genesung von Krankheiten bewirken (vgl. Holt-Lunstead und Uchino 2015, S. 189). Gleichzeitig können diese sozialen Kontakte auch negative gesundheitliche Folgen haben, bspw. wenn sich, wie in der Corona-Pandemie, durch Ansteckung und soziale Vergleichsprozesse (z. B. Normen, die die Einhaltung betreffen, vgl. Geber und Friemel 2020) verstärkt Viruserkrankungen verbreiten (vgl. Cruwys et al. 2020, S. 584). Etablierte Theorien zur Erforschung des Gesundheitsverhaltens (für einen Überblick siehe Faselt et al. 2010), wie die sozial-kognitive Theorie (vgl. Bandura 2004, S. 149), die Theorie des geplanten Verhaltens (vgl. Ajzen 1991, S. 188) oder die sozio-ökologischen Modelle (vgl. Golden und Earp 2012, S. 364), nennen dementsprechend Variablen des sozialen Umfelds als wichtige Determinanten für gesundheitsbezogenes Verhalten.

Allerdings finden diese Determinanten in der medialen Berichterstattung über zentrale Gesundheitsthemen wie Diabetes und Depression (vgl. Reifegerste et al. 2021; Wiedicke et al. 2020) sowie in der kommunikationswissenschaftlichen Erforschung dieser gesundheitsbezogenen Berichterstattung (vgl. Temmann et al. 2019) kaum Beachtung. Dies zeigt sich vor allem bei *Responsibility Frames*, also solchen Frames bzw. Betonungen in der Berichterstattung, die eine bestimmte Verantwortungszuschreibung für Ursachen, Lösungen oder Lösungshindernisse^1^ eines Problems enthalten bzw. nahelegen (vgl. Semetko und Valkenburg 2000, S. 96). Sowohl Inhaltsanalysen (zur Untersuchung der Medienframes in der Berichterstattung) als auch Experimentalstudien (zur Untersuchung der Framing-Effekte) beleuchten bislang vor allem die Verantwortungszuschreibungen auf der individuellen und der gesellschaftlichen Ebene (vgl. Temmann et al. 2019). Dementsprechend finden sich speziell für die Verbreitung und Wirkung von Responsibility Frames auf der Netzwerkebene bis heute nur sehr vereinzelte Aussagen in der Forschung. Inhaltsanalysen zum Responsibility Framing chronischer Erkrankungen zeigen allerdings, dass die Verantwortungsattributionen auf der Netzwerkebene durchaus in der Medienberichterstattung vorhanden sind (vgl. Wiedicke et al. 2020) und auch Effekte auf die Wahrnehmung von Verantwortungsattributionen haben (vgl. Temmann et al. 2020).

Das Responsibility Framing auf der Netzwerkebene in der Berichterstattung könnte demzufolge auch die Wahrnehmung und den Umgang mit Betroffenen und deren Symptomen sowie die Bewältigungsstrategien der Betroffenen selbst beeinflussen. Darauf deuten zumindest die Ergebnisse der Analyse des Responsibility Framing auf gesellschaftlicher und individueller Ebene hin (vgl. Sun et al. 2016, S. 139). Wenn gesellschaftliche Veränderungen als Ursache und Lösung in der Berichterstattung in den Hintergrund treten, nehmen Rezipierende Krankheiten und Krankheitsverläufe verstärkt als individuell verursacht wahr (vgl. Lundell et al. 2013, S. 1122). Außerdem legen die Annahmen der Attributionstheorie (vgl. Weiner 2006, S. 59) nahe, dass bestimmte Eigenschaften des Problems (wie bspw. Kontrollierbarkeit) die Hilfsbereitschaft gegenüber Betroffenen beeinflussen. Allerdings untersuchten vorhandene Studien bislang nicht, inwieweit auch das Framing von Verantwortungszuschreibungen auf der Netzwerkebene Einfluss auf dieses Verhalten hat (vgl. Temmann et al. 2019).

Es lässt sich somit schlussfolgern, dass wichtige Einflussfaktoren des Gesundheitsverhaltens (nämlich die auf der Netzwerkebene) von der kommunikationswissenschaftlichen Erforschung bislang weitgehend ausgeblendet werden. Unsere *Zielstellung* lautet daher, das Konzept der Responsibility Frames um die Ebene sozialer Netzwerke (zusätzlich zur Individual- und Gesellschaftsebene) zu erweitern. Konkret möchten wir aufzeigen, (1) wie Verantwortungszuschreibungen auf der Netzwerkebene in den Framing-Prozess (vgl. Scheufele 1999, S. 106–107, 2003; Chong und Druckman 2007, S. 100f) einbezogen werden können und (2) welche Konsequenzen für die kommunikationswissenschaftliche Forschung sich daraus ergeben. Da es sich bei Responsibility Frames um *generische* (also themenunabhängige) Frames handelt (vgl. Semetko und Valkenburg 2000, S. 97; Dan und Raupp 2018), wird damit eine theoretische Grundlage geschaffen, die sich auf eine Vielzahl weiterer Themen der Kommunikationswissenschaft anwenden lässt. Wir fassen mit dieser Konzeption gleichzeitig die Ergebnisse mehrerer Einzelstudien des DFG-Projektes „Darstellung und Wirkung von Responsibility Frames zu Gesundheitsthemen: Ein Vergleich der Einflussebene des Individuums, des sozialen Netzwerks und der Gesellschaft“ (Projektnummer 404881979) zusammen, um die daraus abgeleiteten Erkenntnisse für andere Themen anschlussfähig zu machen. Es soll daher auch diskutiert werden, (3) welche Themen sich für einen entsprechenden Transfer anbieten.

Einführend stellen wir daher das Responsibility Framing und den Framing Prozess als themenübergreifende Konzepte der Kommunikationswissenschaft sowie den Forschungsstand zum Responsibility Framing der Gesundheitskommunikation vor. Vor dem Hintergrund der Forschung zu sozialen Netzwerken und ihrem (potenziellen) Einfluss auf das Gesundheitsverhalten wird anschließend beleuchtet, welche unterschiedlichen Betrachtungsebenen sich aus dem Zusammenhang von Kommunikation und sozialen Netzwerken ergeben. Auf dieser Grundlage entwickeln wir ein Modell, das die verschiedenen Forschungsstände integriert. Abschließend diskutieren wir mögliche methodische Implikationen für die Forschung zu Responsibility Frames sowie die Frage, für welche weiteren Fragestellungen der Kommunikationswissenschaft die Differenzierung der drei Ebenen sinnvoll erscheint.

## Responsibility Framing

### Theoretische Grundlagen

Trotz der großen theoretischen und paradigmatischen Diversität der Framing-Forschung (vgl. Scheufele und Scheufele 2010, S. 210) werden Frames (weitgehend einheitlich) als Interpretationsmuster betrachtet, die eine sinnvolle Einordnung und effiziente Verarbeitung von Informationen begünstigen (vgl. Scheufele 2004, S. 59). Unterschiedliche Frames können dabei als Ergebnis des „aktiven Prozess[es] des selektiven Hervorhebens von Informationen und Positionen“ (Matthes 2014, S. 10) – dem Framing – verstanden werden.

Als wesentliche Vorgänge innerhalb des Framing-Prozesses nennt Scheufele (1999, S. 117) dabei (1) *Frame Building* und (2) *Frame Setting*, aus denen sich auch die zwei zentralen Stränge der Framing-Forschung^2^ ableiten lassen (vgl. Scheufele und Engelmann 2016, S. 446). Das Framing-Prozess-Modell von Scheufele (1999) baut auf zwei grundsätzlichen Annahmen auf: Erstens, der Differenzierung zwischen individuellen Frames und Medienframes^3^ und zweitens der Unterscheidung zwischen Frames als unabhängiger bzw. abhängiger Variable (vgl. Scheufele 1999, S. 106).

*(1) Frame Building* beschreibt, welche Faktoren die Entstehung von journalistischen Frames beeinflussen; darunter die Kommunikation strategischer Akteur*innen, aber auch strukturelle Gegebenheiten des Mediensystems oder die individuellen Frames der Journalist*innen. Die Medienframes sind in diesem Fall die abhängige Variable. Der Fokus der Forschung liegt somit auf der Kommunikator*innen-Perspektive sowie den Medienframes in journalistischen Beiträgen, was bspw. mit Befragungen und Beobachtungen strategischer Kommunikator*innen bzw. von Journalist*innen im Redaktionsalltag und Inhaltsanalysen der Medienberichterstattung untersucht wird (vgl. Scheufele und Engelmann 2016, S. 446).

Beim (2) *Frame Setting* hingegen bilden die Medienframes die unabhängige Variable. Im Zentrum dieses Prozesses stehen die Wirkungen und somit die Frage, inwiefern (bzw. ob) sich die Medienframes in den individuellen Frames der Rezipient*innen niederschlagen (vgl. Scheufele 1999, S. 117–118). Folglich finden sich in diesem Forschungsstrang vor allem experimentelle Untersuchungen.

Responsibility Frames als Medienframes betonen solche Aspekte eines Themas, die sich auf die Ursachenbewertung („causal interpretation“) und die Verantwortungszuschreibung („treatment recommendation“) für Maßnahmen zur Behebung von Problemen beziehen (Entman 1993, S. 52). Sie lassen sich damit den Betonungsframes zuordnen, wobei in der Gesundheitskommunikation insgesamt häufiger Äquivalenzframes, wie die Gewinn- und Verlustframes, untersucht werden (vgl. Guenther et al. 2020). Um Verantwortungszuschreibungen in der Berichterstattung zu Gesundheitsthemen zu untersuchen, wenden Wissenschaftler*innen vor allem die Analyse thematischer bzw. episodischer Frames nach Iyengar (1990) sowie die Analyse von Responsibility Frames nach Semetko und Valkenburg (2000) an (vgl. Temmann et al. 2019), die ursprünglich für politische Themen entwickelten wurden. Beide gelten als generische, also themenunabhängige bzw. themenübergreifende Frames (vgl. Dan und Raupp 2018).

So nimmt Iyengar (1990, S. 21) an, dass durch einen *episodischen Frame* (d. h. die Darstellung von Einzelpersonen bzw. Einzelschicksalen) die individuelle Verantwortung für ein Problem jedweder Art betont wird. Hingegen lenkt die Journalist*in bei einem *thematischen Frame* den Blick auf ein gesellschaftlich relevantes Thema, das eher strukturelle Probleme aufzeigt. Trotz möglicher Konfundierungen, die sich aus diesen Annahmen ergeben (vgl. Shah et al. 2004), ist das Konzept von Iyengar (1990) mit seiner Unterscheidung von individueller und gesellschaftlicher Ebene der Ursachen und Lösungen nach wie vor das populärste in der Gesundheitskommunikation, um Verantwortungszuschreibungen für Gesundheitsthemen zu untersuchen (vgl. Temmann et al. 2019).

Semetko und Valkenburg (2000, S. 96) beschrieben anhand der Responsibility Frames in der politischen Berichterstattung, ob die Regierung oder Individuen für Probleme oder deren Lösungen verantwortlich sind. Sie hatten in einer Inhaltsanalyse zur politischen Berichterstattung in niederländischen Medien insgesamt fünf weit verbreitete Frames^4^ identifiziert, zu denen auch der Responsibility Frame gehört. Diese Analyselogik wird dementsprechend zunächst überwiegend zur Analyse politischer Kommunikation verwendet. Sofern diese im Gesundheitsbereich überhaupt Anwendung findet, dient sie der Differenzierung zwischen Ursachenzuschreibungen und Handlungsempfehlungen auf der individuellen und der gesellschaftlichen Ebene (vgl. Temmann et al. 2019).

Während die Framing-Konzepte von Iyengar (1990) sowie Semetko und Valkenburg (2000) an den Medieninhalten ansetzen, bietet die *Attributionstheorie* (Weiner 2006) einen sozial-psychologischen Ansatz für die Prozesse der Verantwortungszuschreibung, der sich auf die Framing-Effekte und damit die Wirkung der Medienframes auf die Frames der Rezipient*innen bezieht. Die Attributionstheorie geht davon aus, dass Kausal- und Lösungsattributionen die Basis für verantwortungsbezogene Emotionen und Verhaltensintentionen gegenüber den Betroffenen bilden. Sun et al. (2016, S. 142) entwickelten auf der Basis dieser Annahmen ein *Prozessmodell* der Verantwortungszuschreibung: Rezipierende eines Responsibility Frames mit Attributionen auf gesellschaftlicher Ebene machen dann die Gesellschaft für die Lösung eines Gesundheitsproblems verantwortlich, wenn sie zuvor bereits die Ursache auf der Ebene der Gesellschaft verorteten. So empfinden Menschen weniger Sympathie und Mitgefühl gegenüber Betroffenen, bei denen die Ursache des Gesundheitsproblems als individuell kontrollierbar wahrgenommen wird. Dafür entstehen negative Emotionen wie Wut oder Empörung, und die Hilfsbereitschaft sinkt (vgl. Weiner 2006). Damit benennt Weiner (2006, S. 38–39) nicht nur mögliche emotionale Reaktionen, Einstellungen und Wahrnehmungen, die sich auf die sozialen Kontakte beziehen, sondern auch eine wichtige Verhaltensintention auf der Netzwerkebene (d. h. Lösungen durch das soziale Umfeld) als möglichen Effekt von Medienframes. Allerdings untersuchten Wissenschaftler*innen auch hier bislang nur Medieninhalte mit individueller und gesellschaftlicher Verantwortungsattribution als Einflussfaktoren (vgl. Temmann et al. 2019).

Zum Responsibility Framing existieren somit verschiedene theoretische Grundlagen. Diese lassen nach unserer Einschätzung auch eine Erweiterung des Framing-Konzepts auf die verschiedenen Ebenen der Verantwortungszuschreibung (Individuum, Netzwerk, Gesellschaft) zu. Darauf aufbauende Analysen beschränken sich allerdings zumeist auf das Konzept von Iyengar (1990) und berücksichtigen dementsprechend nur Verantwortungszuschreibungen auf der individuellen und gesellschaftlichen Ebene (vgl. Temmann et al. 2019). Parallel hierzu wird auch die Attributionstheorie, die mögliche Erklärungsansätze für die Wirkung des Frame Settings liefert, in inhaltsanalytischen Studien zu Responsibility Frames immer wieder als theoretische Referenz herangezogen (vgl. Kim et al. 2015, S. 123). Allerdings fehlt dennoch eine explizite Beschreibung der theoretischen Grundlage für experimentelle Forschungsdesigns zum Responsibility Framing auf der Netzwerkebene. Die empirische Prüfung dieser Framing-Effekte steht daher noch weitgehend aus (vgl. Temmann et al. 2019).

### Responsibility Framing in der Gesundheitskommunikation

Aufbauend auf diesen theoretischen Ansätzen zum Framing-Prozess und zum *Responsibility Framing* führten Wissenschaftler*innen zahlreiche Inhaltsanalysen der Medienframes und auch einige experimentelle Studien zur Untersuchung der Effekte auf Rezipient*innenframes durch (vgl. Temmann et al. 2019, siehe Tab. 1).

**Table 1:** 

Einflussebene	Beispiele für Ursachen und Lösungen	Inhaltsanalysen (*n* = 56)	Wirkungsstudien (*n* = 13)
Gesellschaft	Gesetzliche Regelungen, Gesundheitssystem, staatliche Präventionsprogramme	*n* = 55	*n* = 13
Soziales Netzwerk	(Mangelnde) Soziale Unterstützung durch Familie, Einfluss durch Freund*innen, Vorbilder im sozialen Umfeld	*n* = 8	*n* = 0
Individuum	Genetische Determinanten, Lebensstil (Ernährung, Einstellungen, Stress)	*n* = 53	*n* = 13

Anmerkung: *n* gibt die Anzahl der publizierten Studien zum Responsibility Framing bei Gesundheitsthemen zwischen 2004 und 2019 an, die diese Analyseebene explizit berücksichtigen. Details zu Ein- und Ausschlusskriterien sowie der Auswertungsstrategie zu diesem prä-registrierten Systematic Review finden sich unter dem PROSPERO-Eintrag Nr. CRD42020143050

Die vorhandenen *Inhaltsanalysen* zur Darstellung von Gesundheitsthemen wie Depression (vgl. Zhang und Jin 2015, S. 208) und Adipositas (vgl. Kim und Willis 2007, S. 364) in den Printmedien zeigen, dass die Medienberichterstattung überwiegend individuelle (vor allem behaviorale, aber auch medizinisch-biologische) Ursachen und Lösungen präsentieren, während sie gesellschaftlich-politische Einflussfaktoren vernachlässigen. So zeigen etwa Gollust und Lantz (2009, S. 1094) in einer Inhaltsanalyse der Berichterstattung über Diabetes mellitus, dass die vorhandenen Frames die Verantwortung für die Erkrankung mehrheitlich dem Individuum (im Vergleich zu gesellschaftlichen Rahmenbedingungen) zuschreiben und damit im Widerspruch zu Befunden der Gesundheitswissenschaften stehen. Ähnliche Befunde zeigen sich in Untersuchungen zum Framing anderer Gesundheitsthemen wie Übergewicht (vgl. Kim und Willis 2007, S. 372) oder Depression (vgl. Zhang und Jin 2015, S. 216), obwohl auch diese Gesundheitsphänomene wesentlich durch das soziale Netzwerk beeinflusst werden (vgl. DiMatteo 2004, S. 207; Mason et al. 2009, S. 346).

Wenn Wissenschaftler*innen in den Inhaltsanalysen Einflüsse auf das Gesundheitsverhalten untersuchen, die auf der Ebene des sozialen Netzwerks angesiedelt sind, betrachten sie diese meist nicht gesondert, sondern subsumieren sie unter den individuellen oder gesellschaftlichen Einflussfaktoren (vgl. Temmann et al. 2019). So codieren etwa Zhang et al. (2015, S. 212) die Risikofaktoren aus der unmittelbaren sozialen Umwelt, wie Familie, Schule, Arbeitsplatz in der Kategorie der gesellschaftlichen Faktoren für Depression. Dagegen rechneten Mello und Tan (2016, S. 1220) in einer Studie zur Gesundheit von Kindern die Verantwortungszuschreibungen an die Mütter der individuellen Ebene zu. Ebenso haben Kim und Willis (2007, S. 364) in ihrer Studie zum Framing von Übergewicht die negativen Vorbilder im sozialen Umfeld sowie den Besuch einer Selbsthilfegruppe oder von Beratungen als individuelle Ursachen und Lösungen codiert. Hieraus kann geschlussfolgert werden, dass Forschende bislang in der Betrachtung von Responsibility Frames wichtige Aspekte der Medienframes unberücksichtigt ließen.

Auch bei den *experimentellen Untersuchungen* zur Wirkung der Responsibility Frames überwiegt die Unterscheidung zwischen Verantwortungszuschreibungen auf individueller und gesellschaftlicher Ebene. Dies gilt zum einen für die untersuchten Stimuli (vgl. Temmann et al. 2019). Zum anderen zeigt sich die Beschränkung auf die individuellen und gesellschaftlichen Aspekte auch bei den Messvariablen. Das Frame Setting wird überwiegend anhand der Verantwortungszuschreibung, der Zustimmung zu politischen Maßnahmen und Verhaltensintentionen mit Bezug zur Individual- und Gesellschaftsebene untersucht (vgl. Temmann et al. 2019). Lediglich einige wenige Studien haben bereits den Einfluss der Responsibility Frames auf Variablen der Ebene des sozialen Netzwerks festgestellt. Sie zeigen, dass die Verantwortungsframes auf individueller Ebene die Hilfsbereitschaft (vgl. Sun et al. 2016, S. 144) und die Akzeptanz von Hilfe (vgl. Hannah und Cafferty 2006, S. 2997) negativ beeinflussen, während der Verantwortungsframe auf gesellschaftlicher Ebene sie positiv beeinflusst (vgl. Sun et al. 2016, S. 144). Ein weiteres Bespiel für eine abhängige Variable auf Netzwerkebene findet sich bei Jin et al. (2018, S. 63): Die Autor*innen haben untersucht, welche Wirkung Verantwortungsframes auf individueller und gesellschaftlicher Ebene auf die wahrgenommene Selbstwirksamkeit zur Identifikation von Freund*innen oder Familienmitgliedern mit Depression aufweisen. Dabei stellten sie fest, dass der Individualframe zu höherer Selbstwirksamkeit führt als der Gesellschaftsframe. Diese unterschiedlichen Richtungen der Ergebnisse der Einzelstudien machen deutlich, dass es einer Differenzierung der abhängigen Variablen auf den drei Ebenen bedarf, um auch die differenzierten Framing-Effekte der Verantwortungsattributionen zu untersuchen.

## Soziale Netzwerke

### Funktionen

Das Netz sozialer Beziehungen, die ein Individuum umgeben, bildet die Basis für eine Betrachtung der Ursachen und Lösungen auf der Netzwerkebene, da sich aus diesen Beziehungen die potenziellen Ansteckungskontakte, Unterstützer*innen, Vorbilder oder Bewertungsinstanzen speisen (vgl. Valente 2015, S. 205). Dabei beschreibt das soziale Netzwerk die (quantitative) *Struktur*, während sich die (qualitative) *Funktion* der Struktur in sozialer Unterstützung, Meinungsbeeinflussung, Informationsweitergabe etc. ausdrückt (vgl. Holt-Lunstead und Uchino 2015, S. 184).

Zunächst gilt es, in der Struktur des Netzwerks die relevanten Akteur*innen (Einzelpersonen oder kollektive Akteur*innen wie Organisationen) zu identifizieren. Diese können von gesellschaftlichen (z. B. Gesundheitssystem, Gesetzgebung, öffentliche Gelder, Verfügbarkeit einer medizinischen Versorgung) oder individuellen Ressourcen (z. B. Kompetenzen) unterschieden werden. Zudem können auch die Beziehungsstrukturen in einem Gesamtnetzwerk als Betrachtungsobjekt dienen, dessen Strukturen möglicherweise auch relevant für die Verantwortungszuschreibung sind (z. B. der Zusammenhalt in einer Schulklasse, Familie oder Abteilung).

Diese Beziehungen zwischen Akteur*innen in einem sozialen Netzwerk lassen sich zudem durch *bestimmte strukturelle Merkmale* beschreiben, wie bspw. den Grad der Formalität. Dieser beschreibt das Maß, in dem soziale Beziehungen innerhalb einer institutionellen Rolle stattfinden (vgl. Heaney und Israel 2008, S. 191). Während bspw. Ärzt*innen oder Pflegekräfte formelle gesundheitsrelevante Rollen aufweisen, übernehmen Angehörige oder Freund*innen ihre Hilfe überwiegend informell. Weitere relevante Merkmale der Beziehungen zwischen einzelnen Akteur*innen sind u. a. die Beziehungsstärke, die Ähnlichkeit oder die Distanz in einem Netzwerk (vgl. Valente 2015, S. 210).

Soziale Netzwerke können sich auf die Gesundheit sowohl positiv als auch negativ, direkt wie auch vermittelt auswirken und sind damit neben den individuellen und gesellschaftlichen Faktoren wichtige Determinanten von Gesundheit (vgl. Richter und Hurrelmann 2018, Absatz 3). Neben der Valenz und der Direktheit der Effekte kann man auch die Art der Funktion der sozialen Beziehung für die Gesundheit differenzierter betrachteten. So sieht Rook (2015, S. 45) etwa die soziale Unterstützung, die Gemeinschaft und die soziale Kontrolle als positive gesundheitsrelevante Beziehungsfunktionen, während sie Unterstützungsverweigerung, Ablehnung und Bevormundung als negative Formen bezeichnet. Entsprechend sind bei der Analyse der Responsibility Frames zu sozialer Unterstützung neben den dargestellten positiven Effekten auch die negativen Wirkungen zu berücksichtigen. Ähnliche Unterscheidungen finden sich bei Heaney und Israel (2008, S. 191) und Thoits (2011, S. 153), die zusätzlich den sozialen Einfluss, die Selbstwertstärkung und das soziale Kapital als gesundheitsrelevante Funktionen sozialer Netzwerke nennen. Im medialen Kontext erscheinen vor allem drei *Formen sozialer Unterstützung* relevant: (1) Die informationelle Unterstützung, die vor allem die Weitergabe von Informationen zur Lösung der Belastungssituation beschreibt, (2) die emotionale Unterstützung, also die allgemeine Zuneigung, Fürsorge und Anteilnahme an der Belastungssituation, und (3) die instrumentelle Unterstützung, die sich meist in konkreten Dienstleistungen wie Transport oder Hausarbeit ausdrückt (vgl. Lin et al. 2015, S. 372).

Allerdings lassen sich die Ursachen und Lösungen auf den drei Ebenen nicht als völlig voneinander unabhängig betrachten. Vielmehr treten dem sozial-ökologischen Ansatz (vgl. Golden und Earp 2012, S. 364) folgend *Interaktionen* zwischen der Individual‑, Netzwerk-, und Gesellschaftsebene auf. So zeigt etwa eine Studie von Osborn & Egede (2012, S. 249), dass soziale Unterstützung (auf der Netzwerkebene) einen wichtigen Beitrag zur Medikamenteneinnahme (auf der Individualebene) während der Behandlung von Diabetes mellitus bei zusätzlicher Depression leistet. Das spricht dafür, dass therapiebegleitende Maßnahmen, wie soziale Unterstützung, den medizinischen Behandlungserfolg steigern können. Daraus ergibt sich, dass sowohl die Ursachen als auch die Maßnahmen auf individueller, sozialer und gesellschaftlicher Ebene nicht als exklusive Alternativen zueinander auftreten, sondern als unterschiedliche potenzielle Ansatzpunkte, die miteinander verschränkt sein können bzw. Interaktionseffekte aufweisen. Dementsprechend fordern etwa Moran et al. (2016), dass sich die drei Ebenen sowie ihre Interaktionen auch in der Erforschung der Berichterstattung über Gesundheitsthemen widerspiegeln.

### Kommunikation in sozialen Netzwerken vs. Kommunikation über den Einfluss sozialer Netzwerke

In der Kommunikationswissenschaft spielt allerdings nicht nur die Kommunikation innerhalb sozialer Beziehungen als Einflussfaktor auf das Gesundheitsverhalten und die Gesundheit eine wichtige Rolle, sondern auch die öffentliche Kommunikation über eben diese Einflüsse sozialer Beziehungsnetzwerke. Die Kommunikation auf der Ebene des sozialen Netzwerks hat somit in unserer Betrachtung eine doppelte Relevanz und erfolgt auf zwei Betrachtungsebenen. Zunächst können Interaktionen mit sozialen Kontakten (sowohl solche mit als auch solche ohne verbale Kommunikation) Ursache und Lösung (d. h. Einflussfaktor) für gesundheitsbezogenes Verhalten (z. B. das Ernährungsverhalten) oder den Gesundheitszustand (z. B. das psychische Wohlbefinden) sein (zur begrifflichen Komplexität von Gesundheit, Krankheit und Gesundheitsverhalten vgl. Faltermeier 2015; Schnabel und Bödeker 2012, S. 43–45).

So kann unterstützende Kommunikation zur Förderung der Gesundheit anderer in unterschiedlichen Kommunikationsmodi wie Gesprächen, Onlineforen, mobilen Anwendungen oder Sozialen Medien auftreten (vgl. MacGeorge et al. 2011, S. 317). Zwar beruht ein Großteil der prosozialen Handlungen (d. h. solchen, die dazu dienen sollen, anderen zu helfen) auf Face-to-Face-Kommunikation, aber Personen nutzen auch zahlreiche medienvermittelte Modi zum Austausch von emotionaler, informationeller und instrumenteller Unterstützung oder deren Ankündigung (vgl. Stehr 2020, S. 41). Ebenso kann auch die Suche nach Gesundheitsinformation für andere als soziale Unterstützung bzw. gesundheitsrelevantes prosoziales Kommunikationsverhalten betrachtet werden, das die Gesundheit und das Gesundheitsverhalten von anderen (positiv oder negativ) beeinflusst (vgl. Reifegerste und Bachl 2019, S. 393). Allerdings ist Kommunikation (in all ihren Formen, vgl. Baumann und Hurrelmann 2014, S. 10) nur eine Teilmenge von möglichen Einflussfaktoren für Gesundheitsverhalten oder den Gesundheitszustand. Neben unterstützender Kommunikation können bspw. auch Beziehungskonflikte, mangelnde soziale Unterstützung, negative Vorbildfunktionen (z. B. ungesunde Lebensweise in der Familie), Erkrankung eines Angehörigen oder Mobbing am Arbeitsplatz Ursachen für Gesundheitsprobleme auf der Netzwerkebene sein. Lösungsmöglichkeiten sind bspw. soziale Integration, Unterstützungsmöglichkeiten oder Ratgeber*innen im persönlichen Umfeld; sie haben mitunter eine große Bedeutung für die Gesundheit und das Gesundheitsverhalten.

Diese gesundheitsrelevanten Formen der Kommunikation (neben den anderen Determinanten auf der Netzwerkebene) können wiederum als mögliche Ursachen und Lösungen auch Inhalt von Kommunikation sein. Auf dieser zweiten Betrachtungsebene der Metakommunikation findet sich das hier formulierte Anliegen, das Medien-Framing von Verantwortungszuschreibungen auf der Netzwerkebene näher zu beleuchten. Dementsprechend wird möglicherweise die Kommunikation mit Familienangehörigen (als Einflussfaktor auf der Meso-Ebene) als wichtiger Bestandteil für die Behandlung von Depression in den Massenmedien auf der Makroebene (vgl. Chaffee und Berger 1987, S. 143) thematisiert. Die Fragestellung ordnet sich damit in Fragen nach der medialen Konstruktion von Gesundheit und Krankheit ein, genauer: in die kommunikationswissenschaftlichen Fragen nach den Medieninhalten und deren Wirkungen (vgl. Rossmann 2016, S. 301).

## Drei-Ebenen-Modell des Responsibility Framing Prozesses

Nachdem wir die theoretischen Grundlagen und den Forschungsstand aufgezeigt haben, folgt nun die Verknüpfung der Konzepte zu einem integrierten Drei-Ebenen-Modell des Responsibility-Framing-Prozesses. Da soziale Beziehungen (d. h. die verschiedenen Akteur*innen und ihre Funktionen) eine hohe Relevanz für Ursachen und Lösungen zahlreicher in den Medien thematisierter Probleme aufweisen, wird das soziale Netzwerk als eigenständige Betrachtungsebene in den Prozess des Responsibility Framing, d. h. sowohl das Frame Building als auch das Frame Setting, integriert. Somit differenzieren wir die Ebene sozialer Netzwerke von der individuellen und gesellschaftlichen Betrachtungsebene und erweitern damit die Sichtweise um eine mittlere Ebene, sodass wir nun die Einflussfaktoren und Wirkungsmechanismen des Framing-Prozesses auf drei Ebenen sowie deren Interaktionen betrachten.

Ursachen und Lösungen auf der Ebene des *Individuums *sind individuelle Einstellungen und Verhaltensweisen. Zu den individuellen Ursachen von Gesundheitsproblemen zählen bspw. demographische Faktoren, die Persönlichkeit oder der Lebensstil (vgl. Zhang und Jin 2015, S. 208). Zentrale*r Akteur*in ist somit die betroffene Person.

Die Framing-Ebene des *Netzwerks* thematisiert die in Abschn. 3.1 genannten Ursachen und Lösungen eines Gesundheitsproblems, die von sozialen Kontakten ausgehen. Dies sind Akteur*innen des sozialen Netzwerks (z. B. Angehörige, Lehrer*innen, Kolleg*innen, Gleichaltrige) und ihre positiven und negativen Funktionen als sozialer Kontakt (z. B. Unterstützung, schädlicher Einfluss, Kapital).

Dabei betont die Framing-Ebene der *Gesellschaft* jene Ursachen und Lösungen, die Gesetzesänderungen, Reformen des Gesundheitssystems, strukturelle Maßnahmen und Veränderungen und gesellschaftliches Umdenken enthalten. Zudem finden sich hier Akteur*innen mit gesellschaftlichen Funktionen bzw. in ihrer Rolle als Vertreter*innen für gesellschaftliche Funktionen. Zur Einflussebene der Gesellschaft zählen somit alle dem Individuum und dem sozialen Netzwerk übergeordneten gesellschaftlichen Strukturen, die entweder institutioneller, ökonomischer, gesetzlicher oder staatlicher Natur sein können oder sich auf gesellschaftliche Normen, Rollen oder Diskurse beziehen (vgl. Richter und Hurrelmann 2018).

Die *Abgrenzung der Ebenen* kann allerdings (wie bereits am konkreten Beispiel der Depression beschrieben, siehe Abschn. 3.1) verschiedene Herausforderungen mit sich bringen. Mitunter entstehen Gesundheitsprobleme durch Ursachen auf mehreren Ebenen oder durch eine Interaktion der Ursachen auf verschiedenen Ebenen. So hat sich in einer Inhaltsanalyse der Medienberichterstattung über chronische Erkrankungen gezeigt, dass 23 % aller Beiträge die Ursachen und 27 % aller Beiträge die Lösungen einer Interaktion der verschiedenen Ebenen zuschreiben (vgl. Wiedicke et al. 2020).

**Figure 1:**
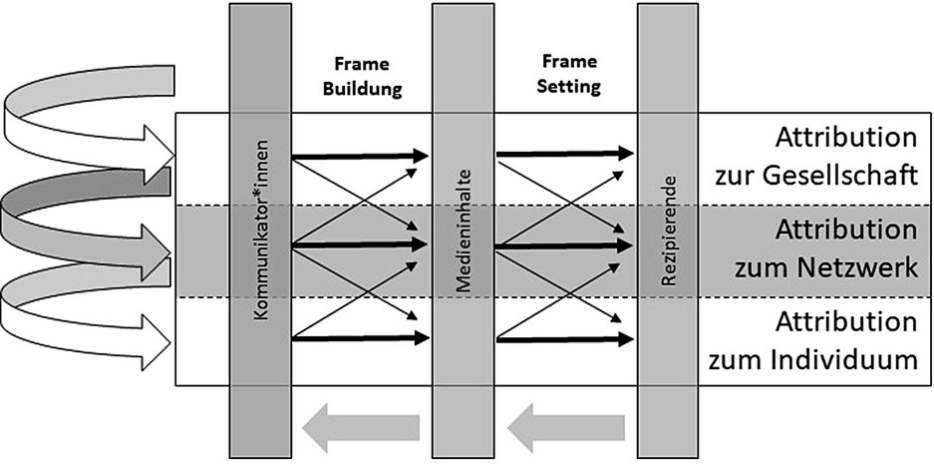

Diese drei Einflussebenen der Verantwortungsattributionen – Individuum, soziales Netzwerk sowie Gesellschaft – sowie mögliche Interaktionen zwischen diesen Einflussebenen, können wir nun in den Framing-Prozess integrieren (siehe Abb. 1).

### Frame Building

Aufgrund der Studien zum Frame Building (vgl. Matthes 2014, S. 52–54) können wir annehmen, dass eine strategische Betonung bestimmter Frame-Ebenen auch zur Betonung dieser Ebenen in den Medienframes führt. Wenn Determinanten auf der Netzwerkebene eine zentrale Rolle für das entsprechende Gesundheitsverhalten spielen, so spiegelt sich dies auch im strategischen Framing der darauf abzielenden Kommunikationsmaßnahmen, z. B. in Form sozialer Appelle wider (vgl. Guttman et al. 2016, S. 910). So sind (neben individuellem Verhalten und gesellschaftlichen Strukturen) auch gesundheitsrelevante (pro-)soziale Handlungen (und die damit ggf. verbundenen Kommunikationsprozesse) das Ziel bzw. der Inhalt von strategischer Kommunikation, wie etwa Aufklärungskampagnen (vgl. Stehr 2019, S. 543). Zum Beispiel zielten zahlreiche Aufrufe der Gesundheitsbehörden in der Corona-Krise auf den sozialen Zusammenhalt und die Abstandsregeln (d. h. Ursachen und Lösungen auf der Netzwerkebene), die sich dann wiederum auch in der journalistischen Berichterstattung niederschlugen (z. B. Brost 2020).

Die Frames von Journalist*innen und ihre Verantwortungsattributionen untersuchen Wissenschaftler*innen allerdings im Vergleich zu den Medienframes bzw. den Frame-Effekten vergleichsweise selten (vgl. Scheufele und Engelmann 2016, S. 446). So berufen sich Ausführungen zu journalistischen Frames (vgl. Scheufele 2003 oder auch Scheufele und Engelmann 2016) auf die Arbeiten von Tuchman (1978) bzw. Gitlin (1980), die jeweils auf die Bedeutung journalistischer Routinen bzw. redaktioneller Diskurse als Einflussvariablen bei der Herausbildung journalistischer Frames aufzeigen, sowie den Aufsatz von Fishman (1978), der sich mit den etablierten Bezugsrahmen von Journalist*innen bei der Einordnung eines Themas auseinandersetzt.

Auf dieser Basis erscheint eine explorative Herangehensweise angebracht. Es stellen sich die Fragen, wem Journalist*innen persönlich die Verantwortung für bspw. die Ursachen und Lösungen bestimmter Gesundheitsprobleme zuschreiben, inwiefern sich diese individuellen Verantwortungsattributionen in ihren Texten widerspiegeln sowie durch welche Faktoren (bspw. redaktionelle Linie, Gespräche mit Kolleg*innen etc.) dieser „Transfer“ beeinflusst wird.

Eine Inhaltsanalyse zum Responsibility Framing von Diabetes mellitus und Depression (vgl. Wiedicke et al. 2020) zeigt allerdings, dass sich auch Determinanten sozialer Netzwerke auf das Gesundheitsverhalten und die Gesundheit in den Medieninhalten auffinden lassen. Zwar dominiert der Responsibility Frame, der die individuelle Verantwortungszuschreibung in den Blick nimmt. Dennoch wird die ursächliche Verantwortung für Depressionen bzw. Diabetes in mehr als einem Viertel der Beiträge auf Netzwerkebene gesehen; während Verantwortung für Lösungen bzw. Lösungshindernisse in mehr als einem Drittel der Beiträge auf Netzwerkebene zugeschrieben wird. Bei den Kausalattributionen überwiegen diese sogar gegenüber Verantwortungszuschreibungen auf der Gesellschaftsebene (vgl. Wiedicke et al. 2020).

Die Bedeutung des sozialen Netzwerks als Einflussebene in der medialen Berichterstattung zeigt sich auch aktuell im Kontext der Berichterstattung zur Corona-Pandemie bzw. der Eindämmung von Covid-19-Infektionen. So ordnet bspw. die *Frankfurter Allgemeine Zeitung* die ursächliche Verantwortung für die Ansteckung von Kindern deren sozialem Netzwerk, d. h. ihren Eltern, zu (vgl. Müller-Jung 2020) und nennt die Familien auch als Verantwortliche für die Bewältigung der Krise (vgl. Brühl 2020, S. 7).

### Frame Setting

Um Wirkungen von Responsibility Frames auf das Framing der Rezipient*innen zu untersuchen, sollten experimentelle Studien auch Medienframes berücksichtigen, die Ursachen und Lösungen auf der Netzwerkebene verorten. Neben einer Ausdifferenzierung der Framing-Stimuli (und damit der Medienframes als unabhängiger Variablen) erscheint es auch notwendig, *die abhängigen Variablen*, d. h. die Betrachtung der Wirkungen, auszuweiten. Wir vermuten, dass dann die Rezipierenden die Ursachen und Lösungen (z. B. Behandlungsoptionen) auch eher den Akteur*innen auf der jeweiligen Ebene zuschreiben. Eine Wirkungsuntersuchung für *Frames auf der Ebene des sozialen Netzwerks* im Vergleich mit den Frames auf den anderen Ebenen steht allerdings noch weitgehend aus. In einer ersten Experimentalstudie zeigt sich, dass Frames, die die Verantwortung dem sozialen Netzwerk zuschreiben, eine signifikant höhere Attribution zum sozialen Netzwerk auslösen als Frames, die die Verantwortung dem Individuum zuschreiben (vgl. Temmann et al. 2020).

Aufbauend auf den in Abschn. 2.1 ausgeführten Annahmen der Attributionstheorie (vgl. Weiner 2006) nehmen wir weitergehend an, dass bei entsprechendem Framing der Ursachen und Lösungen auf der Netzwerkeben die eigene Unterstützungsbereitschaft und die wahrgenommene Unterstützungsbereitschaft durch andere höher ausfällt als bei Medienbotschaften, in denen die Verantwortung auf gesellschaftlicher oder individueller Ebene betont wird, z. B. die selbsteingeschätzte Fähigkeit, Erkrankungen bei anderen zu erkennen (vgl. Jin et al. 2018). Denn Verantwortungszuschreibungen enthalten entsprechend Weiners Attributionstheorie Hinweise auf die Ursache eines Problems, die sich sowohl auf die wahrgenommenen Lösungen als auch auf Verhaltensintention bzw. Verhalten auswirken können (vgl. Sun et al. 2016, S. 142).

Aus der Attributionstheorie lassen sich zudem wichtige Mediatoren und Moderatoren auf der Netzwerkebene für die Untersuchung von Framing-Effekten ableiten, wie bspw. Schuldzuweisungen (vgl. Barry et al. 2013, S. 327), soziale Emotionen wie Sympathie, Mitgefühl oder Wut (vgl. Major 2009, S. 180), soziale Verantwortung (vgl. Bierhoff 2000), Normorientierung, Isolationstendenzen oder Extraversion (vgl. Zerssen und Petermann 2012). Zusätzlich könnte auch die wahrgenommene Beziehungsqualität, d. h. die empfundene Nähe zum*r Betroffenen, eine wichtige Moderatorvariable sein, da sie auch für andere Hilfeleistungen ein wichtiger Prädiktor ist (vgl. Feng und Magen 2016, S. 756).

## Diskussion

Vor dem Hintergrund der Bedeutung des sozialen Netzwerkes für die Medienberichterstattung über Gesundheitsthemen bestand das Ziel des vorliegenden Beitrags darin, das Konzepte des *Responsibility Framing *explizit um die Ebene des *sozialen Netzwerkes* zu erweitern.

Eine Sichtweise, die Verantwortung *entweder* dem Individuum *oder* aber der Gesellschaft zuschreibt, vergibt die Chance darauf, Einflussfaktoren und Wirkmechanismen auf einer Ebene zu untersuchen, auf der nachweislich zahlreiche Ursachen und Lösungen für die Probleme angesiedelt sind. Unser Beitrag liefert daher eine theoretische Grundlage dafür, die Bedeutung des sozialen Netzwerks noch stärker als bisher in der kommunikationswissenschaftlichen Framing-Forschung – sowohl bei der Analyse des Frame Building als auch des Frame Setting – zu berücksichtigen. Dies wird ergänzt um die Möglichkeit, in der Framing-Forschung auch Darstellungen von Ursachen und Lösungen für Themen zu untersuchen, die sich nicht immer klar einer einzelnen Ebene zuschreiben lassen und somit als Interaktionsframes auftreten.

### Anwendungsbereiche in der Kommunikationswissenschaft

Die Relevanz sozialer Netzwerke und ihre Interaktion mit den anderen Ebenen zeigt sich aber nicht nur im Bereich der Gesundheitsforschung, sondern findet sich auch in anderen Themenfeldern in der Kommunikationswissenschaft, wie Politik, Bildung oder Umwelt. So fordern Kommunikator*innen etwa in der #BlackLivesMatter-Bewegung *weiße* Personen dazu auf, sich solidarisch mit People of Color in ihrem sozialen Umfeld zu zeigen. Einstellungen der Eltern und der Gleichaltrigen beeinflussen weiterhin ausländerfeindliche Einstellungen sowie die politische Teilhabe von Jugendlichen in erheblichem Ausmaß (vgl. Schmid 2008; Böhm-Kasper 2006). Ebenso bestimmen häufig Meinungsführende im persönlichen wie auch digitalen sozialen Umfeld die politischen Einstellungen (vgl. Geise 2017), während die Bildung wesentlich vom Elternhaus und Gleichaltrigen abhängt (vgl. Hurrelmann et al. 2011, S. 324). Auch Gespräche mit anderen Kontakten des sozialen Umfelds (face-to-face oder medial vermittelt) stellen im Sinne einer Anschlusskommunikation einen wichtigen Einflussbereich für z. B. politische Einstellungen dar (vgl. Gehrau 2019). Allerdings ist bislang weitgehend unklar, inwieweit diese Einflussfaktoren auf der Netzwerkebene auch in der Medienberichterstattung vorkommen und welchen Medieneffekt sie haben.

Da es sich bei Responsibility Frames um *generische* (also themenunabhängige) Frames handelt (vgl. Dan und Raupp 2018, S. 206), lassen sie sich auf vielfältige weitere Themen der Kommunikationswissenschaft anwenden. Über den Bereich der Gesundheitskommunikation hinaus hat somit das Framing von Ursachen und Lösungsansätzen auf der Netzwerkebene nach unserer Einschätzung auch Relevanz für Themen wie soziale Ungleichheit (Armut, Bildung), Umweltthemen (Klimawandel, Naturschutz) und politische Kommunikation (Integration, Meinungsbildung, politisches Interesse und Teilhabe). Folglich scheint es bei Themen, die zahlreiche Einflussfaktoren auf der Netzwerkebene aufweisen, angebracht, diese Ebene in die Framing-Analysen einzubeziehen. Ziel kann dabei bspw. sein, zu untersuchen, inwieweit Aktivist*innen, Journalist*innen sowie Rezipient*innen diese Ebene wahrnehmen und welche Auswirkungen dies auf ihr Verhalten hat. Konkret könnten Kommunikationswissenschaftler*innen bspw. prüfen, inwieweit die Darstellung von Ursachen und Lösungen durch das soziale Umfeld (z. B. Ansteckung bei Freund*innen vs. Abstand halten zu Freund*innen) auch entsprechende Wirkungen auf Verantwortungsattributionen oder Verhaltensintentionen (z. B. Fernbleiben) hervorruft.

Dies entspricht auch allgemeineren Forderungen in der Kommunikationswissenschaft nach einer stärkeren Berücksichtigung der Meso-Ebene, die eine Netzwerkperspektive und die Berücksichtigung sozialer Interaktionen für zunehmend notwendig erachten, um die Komplexität und Dynamik von Kommunikation im digitalen Medienwandel zu untersuchen (vgl. Waldherr 2017; Neuberger 2014; Strippel et al. 2018). Zudem integrieren wir damit Konzepte der interpersonalen Kommunikation und relationale Perspektiven in etablierte Konzepte der öffentlichen Massenkommunikation und leisten einen Beitrag zur Differenzierungs- und Theoriearbeit, was wiederum auch im Hinblick auf die Weiterentwicklung der Kommunikationswissenschaft gefordert wird (vgl. Theis-Berglmair 2016, S. 387; Klinger 2018, S. 246).

Kommunikationswissenschaftlichen Studien kommt demnach in den Sozialwissenschaften eine tragende Rolle zu, da sie Phänomene auf mehreren Ebenen (Mikro-Meso-Makro) gleichzeitig berücksichtigen. So können sie betrachten, wie sich individuelles Verhalten in sozialen Interaktionen (d. h. auf der Meso-Ebene) etabliert und welche Folgen sich daraus für individuelles Verhalten sowie für übergeordnete gesellschaftliche Makro-Phänomene entwickeln (vgl. Quandt und Scheufele 2012). Dementsprechend sollte sich diese differenzierte Betrachtungsweise des Faches auch in der Analyse von Frames (als einem zentralen Konzept der Kommunikationswissenschaft) widerspiegeln.

Übergreifende Analysen der Framing-Forschung in der Gesundheitskommunikation (vgl. Dan und Raupp 2018; Guenther et al. 2020), die das Responsibility Framing neben anderen Framing-Konzepten wie dem Gain-Loss-Framing in die psychologische und soziologische Medienforschung einordnen, machen zudem deutlich, welche weiteren Forschungsfelder einer genaueren Analyse bedürfen. So bieten sich bspw. die visuellen Frames nicht nur für den Transfer auf das Konzept der Responsibility Frames, sondern auch für eine Erweiterung um die Ebene des Netzwerks an. Dies deutet zumindest eine Studie zur Wirkung von Gesundheitsappellen mit Gruppenbildern an (vgl. Reifegerste und Rossmann 2017).

### Methodische Implikationen

Zukünftige kommunikationswissenschaftliche Arbeiten sollten daher die Drei-Ebenen-Systematisierung der Responsibility Frames auch in Studien zum Frame Building und Frame Setting differenzieren. Mit Blick auf die unterschiedlichen Probleme und Betroffenengruppen gilt es bspw. themenspezifisch für die Inhaltsanalysen der Medienframes zu entscheiden, welche Akteur*innen und welche Funktionen des sozialen Netzwerks themenspezifisch zu berücksichtigen sind. So kann es bei der Betrachtung eines Gesundheitsproblems von Kindern relevant erscheinen, die Mitschüler*innen, die Eltern und das Schulsetting zu berücksichtigen, während für das politische Engagement von Erwachsenen die Nachbar*innen und Kolleg*innen wichtige Akteur*innen des sozialen Netzwerks darstellen. Möglicherweise ist es für die jeweiligen Themen auch notwendig, die drei Ebenen weiter zu differenzieren und bspw. wie in der Attributionstheorie bei den individuellen Determinanten zwischen eher kontrollierbaren Faktoren (wie der Lebensweise) und eher nicht-kontrollierbaren genetischen Faktoren zu unterscheiden.

Die drei Ebenen der Responsibility Frames könnten darüber hinaus auch in den einzelnen Medien unterschiedlich verteilt sein. Daher könnte die zukünftige Forschung ergänzend untersuchen, in welchen Mediengattungen Responsibility Frames auf der Netzwerkebene häufiger zu finden sind. So zeigen etwa einzelne Studien in *sozialen Online-Netzwerken* wie Twitter (vgl. Cavazos-Rehg et al. 2016) oder Pinterest (vgl. Guidry et al. 2016), dass Unterstützungsbotschaften (d. h. Botschaften, die Lösungen auf der Meso-Ebene darstellen) neben der Symptombeschreibung von Depressionen der häufigste Inhalt sind. Fraglich ist daher auch, inwieweit Webseiten, Foren und soziale Medien, die insbesondere für psychische und chronische Krankheiten eine wichtige Informationsquelle sind (vgl. Link und Baumann 2020), die Frames aus der Berichterstattung übernehmen und inwiefern sie die Relevanz sozialer Beziehungen und sozialer Unterstützung als Behandlungsmöglichkeit ergänzend aufgreifen bzw. darstellen. Darüber hinaus lässt sich medienspezifisch auch betrachten, von welchen Quellen die unterschiedlichen Responsibility Frames stammen (z. B. Journalist*innen, Gesundheitsinstitutionen, Pharmaunternehmen) und welche Darstellungsformen (z. B. Expert*innenaussagen, Statistiken, Fallbeispiele, Bilder) vorkommen. Denn in experimentellen Untersuchungen hat sich bereits gezeigt, dass die Darstellung mit Fallbeispielen sich signifikant auf die Zuschreibung zum Individuum auswirkt (vgl. Barry et al. 2013). Dementsprechend gilt es auch hier das Frame Building und Frame Setting auf allen drei Ebenen der Verantwortungszuschreibung zu prüfen.

Allerdings sollte sich die Analyse nicht grundsätzlich auf die drei Ebenen beschränken, sondern davon ausgehen, dass sich zwischen den zwei standardmäßig im Responsibility Framing angelegten Ebenen Individuum und Gesellschaft weitere Handlungsebenen mit relevanten Ursachen und Lösungen bzw. Lösungshindernissen befinden. So macht etwa das Mehrebenen-Modell der Handlungsebenen von Gesundheitsförderung (vgl. Altgeld 2020) deutlich, dass neben der Handlungsebene Individuen und Gruppen, die Institutionen, das Gemeinwesen und die Politik relevant sind. Themenspezifisch lassen sich hier vermutlich weitere Einfluss- bzw. Handlungsebenen identifizieren bzw. differenzieren, die ggf. im Responsibility Framing Berücksichtigung finden sollten.

## Footnotes

1 Iyengar (1990, S. 28) beschreibt in seiner Ausgangsarbeit zu Responsibility Frames die sogenannte *treatment responsibility* nicht nur als Verantwortung für die Lösung eines bestimmten Problems, sondern auch als Verantwortung für Faktoren, die eine solche Lösung verhindern.

2 Zwei weitere bei Scheufele (1999, S. 117) beschriebene Framing-Prozesse sind individuelle Framing-Effekte sowie Journalist*innen als Publikum.

3 *Individuelle Frames* oder Rezipient*innenframes bezeichnen „an individual’s cognitive understanding of a given situation“ (Chong und Druckman 2007, S. 101), während der Begriff des *Medienframe* sich auf bestimmte Formen in der Berichterstattung bezieht, „that are used to construct news stories and the processes that shape this construction“ (Borah 2011, S. 247).

4 Außer Responsibility Frames zählen auch* Conflict Frame, Human Interest Frame, Economic Consequences Frame *sowie *Morality Frame *dazu.

## References

[CR84] Altgeld T (2020). Wieviel zersplitterte Zuständigkeit verträgt unsere Gesundheit?. Public Health Forum.

[CR1] Ajzen I (1991). The theory of planned behavior. Organizational Behavior and Human Decision Processes.

[CR2] Bandura A (2004). Health promotion by social cognitive means. Health Education & Behavior.

[CR3] Barry CL, Brescoll VL, Gollust SE (2013). Framing childhood obesity: How individualizing the problem affects public support for prevention. Political Psychology.

[CR4] Baumann E, Hurrelmann K, Hurrelmann K, Baumann E (2014). Gesundheitskommunikation: Eine Einführung. Handbuch Gesundheitskommunikation.

[CR5] Bierhoff HW (2000). Skala der sozialen Verantwortung nach Berkowitz und Daniels: Entwicklung und Validierung. Diagnostica.

[CR6] Böhm-Kasper O (2006). Schulische und politische Partizipation von Jugendlichen. Welchen Einfluss haben Schule, Familie und Gleichaltrige auf die politische Teilhabe Heranwachsender?. Diskurs Kindheits-und Jugendforschung.

[CR7] Borah P (2011). Conceptual issues in framing theory: a systematic examination of a decade’s literature. Journal of Communication.

[CR8] Brost, M. (2020, 18. März). Coronavirus und Solidarität: Zusammen – aber wie geht das noch mal? Was es bedeutet, in Zeiten von Corona solidarisch zu sein. *Die Zeit*. Ausgabe Nr. 13. https://www.zeit.de/2020/13/coronavirus-solidaritaet-zusammenhalt-gesellschaft-quarantaene

[CR9] Brühl, M. (2020, 28. Mai). Zuhören im Lockdown. *Frankfurter Allgemeine Zeitung, *S. 7.

[CR10] Cavazos-Rehg PA, Krauss MJ, Sowles S, Connolly S, Rosas C, Bharadwaj M, Bierut LJ (2016). A content analysis of depression-related Tweets. Computers in Human Behavior.

[CR11] Chaffee SH, Berger CR, Berger CR, Chaffee SH (1987). Levels of analysis: an introduction. Handbook of communication science.

[CR12] Chong D, Druckman JN (2007). A theory of framing and opinion formation in competitive elite environments. Journal of Communication.

[CR13] Cruwys T, Stevens M, Greenaway KH (2020). A social identity perspective on COVID-19: health risk is affected by shared group membership. The British journal of social psychology.

[CR14] Dan V, Raupp J (2018). A systematic review of frames in news reporting of health risks: characteristics, construct consistency vs. name diversity, and the relationship of frames to framing functions. Health, Risk & Society.

[CR15] DiMatteo (2004). Social support and patient adherence to medical treatment: a meta-analysis. Health psychology.

[CR16] Entman RM (1993). Framing: toward clarification of a fractured paradigm. Journal of Communication.

[CR17] Faltermeier, T. (2015). Gesundheitsverhalten, Krankheitsverhalten, Gesundheitshandeln. https://www.leitbegriffe.bzga.de/systematisches-verzeichnis/allgemeine-grundbegriffe/gesundheitsverhalten-krankheitsverhalten-gesundheitshandeln/. Zugegriffen: 30. Jan. 2017.

[CR18] Faselt F, Hoffmann S, Hoffmann S, Hoffmann S, Müller S (2010). Theorien des Gesundheitsverhaltens. Gesundheitsmarketing: Gesundheitspsychologie und Prävention.

[CR19] Feng B, Magen E (2016). Relationship closeness predicts unsolicited advice giving in supportive interactions. Journal of Social and Personal Relationships.

[CR20] Fishman M (1978). Crime waves as ideology. Social Problems.

[CR21] Geber, S., & Friemel, T. (2020). *Social Distancing als normatives Verhalten: Wie Normen und Kommunikation das Abstandhalten in der Corona-Krise beeinflussen*. Leipzig: Vortrag auf der Jahrestagung der Fachgruppe Gesundheitskommunikation.

[CR22] Gehrau V (2019). Gespräche über Medien in Zeiten von Mobilkommunikation und sozialen Onlinenetzen. Publizistik.

[CR23] Geise S (2017). Meinungsführer und der „Flow of Communication“.

[CR24] Gitlin T (1980). The whole world is watching. Mass media in the making & unmaking of the New Left.

[CR25] Golden SD, Earp JAL (2012). Social ecological approaches to individuals and their contexts: twenty years of health education & behavior health promotion interventions. Health education & behavior.

[CR26] Gollust SE, Lantz PM (2009). Communicating population health: print news media coverage of type 2 diabetes. Social Science and Medicine.

[CR27] Guenther L, Gaertner M, Zeitz J (2020). Framing as a concept for health communication: a systematic review. Health Communication.

[CR28] Guidry J, Zhang Y, Jin Y, Parrish C (2016). Portrayals of depression on Pinterest and why public relations practitioners should care. Public Relations Review.

[CR29] Guttman N, Siegal G, Appel N, Bar-On G (2016). Should altruism, solidarity, or reciprocity be used as prosocial appeals?. Journal of Communication.

[CR30] Hannah G, Cafferty TP (2006). Attribute and responsibility framing effects in television news coverage of poverty. Journal of Applied Social Psychology.

[CR31] Heaney CA, Israel BA, Glanz K, Rimer BK, Viswanath K (2008). Social networks and social support. Health behavior and health education. Theory, research, and practice.

[CR32] Holt-Lunstead J, Uchino BN, Glanz K, Rimer BK, Viswanath K (2015). Social support and health. Health behavior and health education. Theory, research, and practice.

[CR33] Hurrelmann K, Andresen S, Schneekloth U (2011). Diskurs Kindheits- und Jugendforschung.

[CR34] Iyengar S (1990). Framing responsibility for political issues: the case of poverty. Political behavior.

[CR35] Jin Y, Zhang Y, Lee Y-I, Tang Y (2018). Learn after reading: effects of news framing and responsibility attribution on Chinese college students’ perceived efficacy in identifying others and themselves with depression. Asian Journal of Communication.

[CR38] Kim S-H, Willis A (2007). Talking about obesity: news framing of who is responsible for causing and fixing the problem. Journal of Health Communication.

[CR37] Kim S-H, Tanner AH, Foster CB, Kim SY (2015). Talking about health care: news framing of who is responsible for rising health care costs in the United States. Journal of Health Communication.

[CR39] Klinger U (2018). Aufstieg der Semiöffentlichkeit: Eine relationale Perspektive. Publizistik.

[CR40] Lin T-C, Hsu JS-C, Cheng H-L, Chiu C-M (2015). Exploring the relationship between receiving and offering online social support: a dual social support model. Information & Management.

[CR41] Link E, Baumann E (2020). Nutzung von Gesundheitsinformationen im Internet: personenbezogene und motivationale Einflussfaktoren. Bundesgesundheitsblatt, Gesundheitsforschung, Gesundheitsschutz.

[CR42] Lundell H, Niederdeppe J, Clarke C (2013). Public views about health causation, attributions of responsibility, and inequality. Journal of Health Communication.

[CR43] MacGeorge EL, Feng B, Burleson BR, Knapp ML, Daly JA, Knapp ML, Daly JA (2011). Supportive communication. The SAGE handbook of interpersonal communication.

[CR44] Major LH (2009). Break it to me harshly: The effects of intersecting news frames in lung cancer and obesity coverage. Journal of Health Communication.

[CR45] Mason MJ, Schmidt C, Abraham A, Walker L, Tercyak K (2009). Adolescents’ social environment and depression: social networks, extracurricular activity, and family relationship influences. Journal of clinical psychology in medical settings.

[CR46] Matthes J (2014). Framing.

[CR47] Mello S, Tan ASL (2016). Who’s responsible? Media framing of pediatric environmental health and mothers’ perceptions of accountability. Journal of Health Communication.

[CR48] Moran MB, Frank LB, Zhao N, Gonzalez C, Thainiyom P, Murphy ST, Ball-Rokeach SJ (2016). An argument for ecological research and intervention in health communication. Journal of Health Communication.

[CR49] Müller-Jung, J. (2020, 3. Juni). Kein bisschen Rückzieher. Verbesserte Drosten-Studie. *FAZ.net*.

[CR50] Neuberger C (2014). Konflikt, Konkurrenz und Kooperation: Interaktionsmodi in einer Theorie der dynamischen Netzwerköffentlichkeit. Medien & Kommunikationswissenschaft.

[CR51] Osborn CY, Egede LE (2012). The relationship between depressive symptoms and medication nonadherence in type 2 diabetes: the role of social support. General hospital psychiatry.

[CR52] Quandt T, Scheufele B (2012). Ebenen der Kommunikation. Mikro-Meso-Makro-Links in der Kommunikationswissenschaft.

[CR53] Reifegerste D, Bachl M (2019). Informationssuche als Beziehungstat. Der Zusammenhang zwischen relationalen Faktoren und Motiven der stellvertretenden Suche nach Gesundheitsinformationen. [Information seeking as an act of relationship]. Studies in Communication Media.

[CR54] Reifegerste D, Rossmann C (2017). Promoting physical activity with group pictures. Affiliation-based visual communication for high-risk populations. Health Communication.

[CR55] Reifegerste D, Wiedicke A, Temmann LJ (2021). Medienberichterstattung zu Präventions- und Therapiemöglichkeiten an den Beispielen Diabetes mellitus und Depression. Bundesgesundheitsblatt – Gesundheitsforschung – Gesundheitsschutz.

[CR56] Richter, M., & Hurrelmann, K. (2018): Determinanten von Gesundheit. In: Bundeszentrale für gesundheitliche Aufklärung (Hg.): Leitbegriffe der Gesundheitsförderung und Prävention, Glossar zu Konzepten, Strategien und Methoden: BZGA – Federal Centre for Health Education. Online verfügbar unter https://www.leitbegriffe.bzga.de/alphabetisches-verzeichnis/determinanten-von-gesundheit/.

[CR57] Rook KS (2015). Social networks in later life: weighing positive and negative effects on health and well-being. Current Directions in Psychological Science.

[CR58] Rossmann C, Richter M, Hurrelmann K (2016). Die mediale Konstruktion von Gesundheit und Krankheit. Soziologie von Gesundheit und Krankheit.

[CR59] Scheufele B (2003). Framing – Frames – Framing-Effekte. Theoretische und methodische Grundleguung des Framing-Ansatzes sowie empirische Befunde zur Nachrichtenproduktion.

[CR60] Scheufele B (2004). Framing-Effekte auf dem Prufstand. Eine theoretische, methodische und empirische Auseinandersetzung mit der Wirkungsperspektive des Framing-Ansatzes. Medien & Kommunikationswissenschaft.

[CR63] Scheufele DA (1999). Framing as a theory of media effects. Journal of Communication.

[CR61] Scheufele B, Engelmann I, Löffelholz M, Rothenberger L (2016). Journalismus und Framing. Handbuch Journalismustheorien.

[CR62] Scheufele BT, Scheufele D, D’Angelo P, Kuypers JA (2010). Of spreading activation, applicability, and schemas: conceptual distinctions and their operational implications for measuring frames and Framing Effects. Doing news framing analysis: empirical and theoretical perspectives.

[CR64] Schmid C (2008). Ausländerfeindlichkeit bei Jugendlichen. Manifester und latenter politischer Sozialisationseinfluss des Elternhauses und der Einfluss befreundeter Gleichaltriger. Zeitschrift für Pädagogik.

[CR65] Schnabel P-E, Bödeker M (2012). Gesundheitskommunikation.

[CR66] Semetko HA, Valkenburg PM (2000). Framing European politics: a content analysis of press and television news. Journal of Communication.

[CR67] Shah DV, Kwak N, Schmierbach M, Zubric J (2004). The interplay of news frames on cognitive complexity. Human Communication Research.

[CR68] Stehr P, Rossmann C, Hastall MR (2019). Prosoziales Handeln und Gesundheit aus Sicht der Kommunikationswissenschaft. Handbuch der Gesundheitskommunikation. Kommunikationswissenschaftliche Perspektiven.

[CR69] Stehr P, Gehrau V, Waldherr A, Scholl A (2020). Prosoziales Handeln in unterschiedlichen Kommunikationsmodi: Ergebnisse einer teilstandardisierten Tagebuchstudie. Integration durch Kommunikation. Jahrbuch der Publizistik- und Kommunikationswissenschaft 2019.

[CR70] Strippel C, Bock A, Katzenbach C, Mahrt M, Merten L, Nuernbergk C, Pentzold C, Puschmann C, Waldherr A (2018). Die Zukunft der Kommunikationswissenschaft ist schon da, sie ist nur ungleich verteilt. Publizistik.

[CR71] Sun Y, Krakow M, John KK, Liu M, Weaver J (2016). Framing obesity. How rews frames shape attributions and behavioral responses. Journal of Health Communication.

[CR73] Temmann, L. J., Wiedicke, A., Schaller, S., Reifegerste, D., & Scherr, S. (2019). *Responsibility frames in health communication: a systematic review of their representation and effects. *Zürich: Vortrag auf der Jahrestagung der TWG Health Communication und der Fachgruppe Gesundheitskommunikation.

[CR72] Temmann LJ, Wiedicke A, Reifegerste D, Scherr S (2020). Wer erkrankt, hat es sich selbst zuzuschreiben? Verantwortungsattribution als Wirkung von Responsibility Frames in der Gesundheitsberichterstattung.

[CR74] Theis-Berglmair AM (2016). Auf dem Weg zu einer Kommunikationswissenschaft. Publizistik.

[CR75] Thoits PA (2011). Mechanisms linking social ties and support to physical and mental health. Journal of health and social behavior.

[CR76] Tuchman G (1978). Making news. A study in the construction of reality.

[CR77] Valente TW, Glanz K, Rimer BK, Viswanath K (2015). Social networks and health behaviors. Health behavior and health education. Theory, research, and practice.

[CR78] Waldherr A (2017). Öffentlichkeit als komplexes System. Theoretischer Entwurf und methodische Konsequenzen. Medien & Kommunikationswissenschaft.

[CR79] Weiner B (2006). Social motivation, justice, and the moral emotions. An attributional approach.

[CR80] Wiedicke, A., Reifegerste, D., Temmann, L. J., & Scherr, S. (2020). *Who’s causing and fixing diabetes—me, we or both? The portrayal of responsibility in media coverage*. Gold Coast. Vortrag auf der Jahrestagung der International Communication Association

[CR81] von Zerssen D, Petermann F (2012). Münchner Persönlichkeitstest: MPT.

[CR82] Zhang Y, Jin Y (2015). Who’s responsible for depression?. The Journal of International Communication.

[CR83] Zhang Y, Jin Y, Tang Y (2015). Framing depression: cultural and organizational influences on coverage of a public health threat and attribution of responsibilities in Chinese news media, 2000–2012. Journalism & Mass Communication Quarterly.

